# Nlp promotes autophagy through facilitating the interaction of Rab7 and FYCO1

**DOI:** 10.1038/s41392-021-00543-1

**Published:** 2021-04-16

**Authors:** Wenchang Xiao, Danna Yeerken, Jia Li, Zhangfu Li, Lanfang Jiang, Dan Li, Ming Fu, Liying Ma, Yongmei Song, Weimin Zhang, Qimin Zhan

**Affiliations:** 1 State Key Laboratory of Molecular Oncology, National Cancer center/National Clinical Research Center for Cancer/Cancer Hospital, Chinese Academy of Medical Sciences and Peking Union Medical College, Beijing, China; 2 Department of Biomedical Engineering, College of Life Science and Technology, Huazhong University of Science and Technology (HUST), Wuhan, China; 3 Key Laboratory of Carcinogenesis and Translational Research (Ministry of Education/Beijing), Laboratory of Molecular Oncology, Peking University Cancer Hospital & Institute, Beijing, China; 4 Department of Hepato-Pancreato-Biliary Surgery, Peking University Shenzhen Hospital, Shenzhen Peking University-The Hong Kong University of Science and Technology Medical Center, Shenzhen, China; 5 Research Unit of Molecular Cancer Research, Chinese Academy of Medical Sciences, Beijing, China; 6 Institute of Cancer Research, Shenzhen Bay Laboratory, Shenzhen, China

**Keywords:** Oncogenes, Cancer models

## Abstract

Autophagy is the main degradation pathway to eliminate long-lived and aggregated proteins, aged or malfunctioning organelles, which is essential for the intracellular homeostasis and prevention of malignant transformation. Although the processes of autophagosome biogenesis have been well illuminated, the mechanism of autophagosome transport remains largely unclear. In this study, we demonstrated that the ninein-like protein (Nlp), a well-characterized centrosomal associated protein, was able to modulate autophagosome transport and facilitate autophagy. During autophagy, Nlp colocalized with autophagosomes and physically interacted with autophagosome marker LC3, autophagosome sorting protein Rab7 and its downstream effector FYCO1. Interestingly, Nlp enhanced the interaction between Rab7 and FYCO1, thus accelerated autophagic flux and the formation of autophagolysosomes. Furthermore, compared to the wild-type mice, *NLP* deficient mice treated with chemical agent DMBA were prone to increased incidence of hepatomegaly and liver cancer, which were tight associated with the hepatic autophagic defect. Taken together, our findings provide a new insight for the first time that the well-known centrosomal protein Nlp is also a new regulator of autophagy, which promotes the interaction of Rab7 and FYCO1 and facilitates the formation of autophagolysosome.

## Introduction

Autophagy is a catabolic process by which eukaryotic cells portions of their own cytoplasm,^1^ such as protein aggregates, aged or even malfunctioning organelles.^2^ It can be induced by nutrition deficiency and other cell stress conditions to maintain cell homeostasis.^3^ Autophagy is commonly described as the processes during which vesicles containing cytosolic cargo, called autophagosomes, are transported to lysosomes and fuse, then to form the so-called autophagolysosomes,^4^ in which the degradation finally happens.

In the cytosol, the components to be degraded are engulfed by a membrane sack called the phagophore or the isolation membrane. Then the autophagosome is formed once the phagophore is sealed to be a closed, double-membrane vesicle.^5^ Genetic screens in yeast have identified a group of autophagy-related proteins (ATG), which are mostly involved in the formation of autophagosomes.^6^ Once an autophagosome is formed, it is transport to the cortex area in cytosol, there autophagosome is fused with lysosome to form an autophagolysosome.^7^ Autophagolysosome is considered to be the final modality of vesicles containing cellular waste cargo.^8^ The processes of the formation and degradation of autophagosome are well elucidated. However, the transportation of autophagosome in the cytosol remains largely unclear.

The transport network of cellular vesicle is regulated by a group of proteins. RAS-related GTP-binding proteins (Rab protein family) are GTPases that recruit and activate several proteins involved in membrane fusion and vesicle transport.^9^ It’s reported that Rab5,^10^ Rab7,^11^ Rab11,^12^ Rab24,^13^ Rab32,^14^ and Rab33B^15^ are involved in autophagy. Especially, Rab7 plays essential roles in vesicle sorting and fate decision.^16^ Rab7 locates in endosome, lysosome, and autophagosome.^17^ Rab7 is considered to recruit two main proteins as its downstream effectors—FYVE and coiled-coil domain-containing 1 (FYCO1)^18^ and Rab interaction lysosomal protein (RILP).^19^ FYCO1 binds to Rab7 and promotes microtubule (MT) plus end-directed transport of Rab7 labeled autophagosome.^20^ FYCO1 is also a multidomain autophagy adaptor protein interacting with autophagosomal membrane components MT-associated protein 1 light chain 3 (LC3) and phosphatidylinositol 3-phosphate (PI3P) via its C-terminal LC3-interacting region (LIR) motif.^18^ This process is required for the efficient maturation of autophagosomes during basal autophagy.^21^

MT is the basal compartment of the cell skeleton and the main track for organelles distribution and vesicle transport.^21^ The major MT organizing center of mammalian cells is centrosome.^22^ Centrosome along with its associated compartmental proteins serve as the key regulators and promoters of mitosis,^23^ which has been illuminated widely. Nlp (Ninein-like protein) is a novel centrosome protein that is implicated in centrosome maturation by recruiting γ-tubulin ring complexes (γ-TuRCs) during interphase.^24^ It plays an essential role in the whole processes of mitosis as a substrate of several key mitotic kinases including Plk1,^25^ Nek2,^26^ Cdc2,^27^ and Aurora B.^28^ Nlp is also recently identified as MT binding protein, it binds to γ-tubulin and hGCP4 in G2/M phases to regulate centrosome maturation, MT anchoring, and organization. Overexpression of Nlp leads to its continuous locating on centrosome, prevention of MT organization and spindle formation, which results in abnormal spindle.^25^ Despite the known functions during mitosis, Nlp shows its potential function in several processes of vesicle transport. The overexpression of Nlp induces the fragmentation of the Golgi, and cause lysosomes to disperse toward the cell periphery in interphase.^29^ In the process of ciliopathies, Nlp associates with CC2D2A and functions in Rab8-MICAL3-regulated vesicle trafficking.^30^

In this study, we demonstrated that Nlp was involved in the regulation of autophagy. Nlp colocalized with autophagy-related proteins ATG5, ATG16L1, LC3, and LAMP2. Importantly, our results revealed that Nlp physically interacted with the key vesicle sorting protein Rab7 and its downstream effector FYCO1, which participated in the determination of autophagosome movement direction along MT. Nlp promoted the interaction between Rab7 and FYCO1, thus enhancing autophagosome fusion with the lysosome. Collectively, these findings have extended our understanding on how autophagosome transports toward lysosome.

## Results

### Nlp colocalizes with ATG

**Fig. 1:**
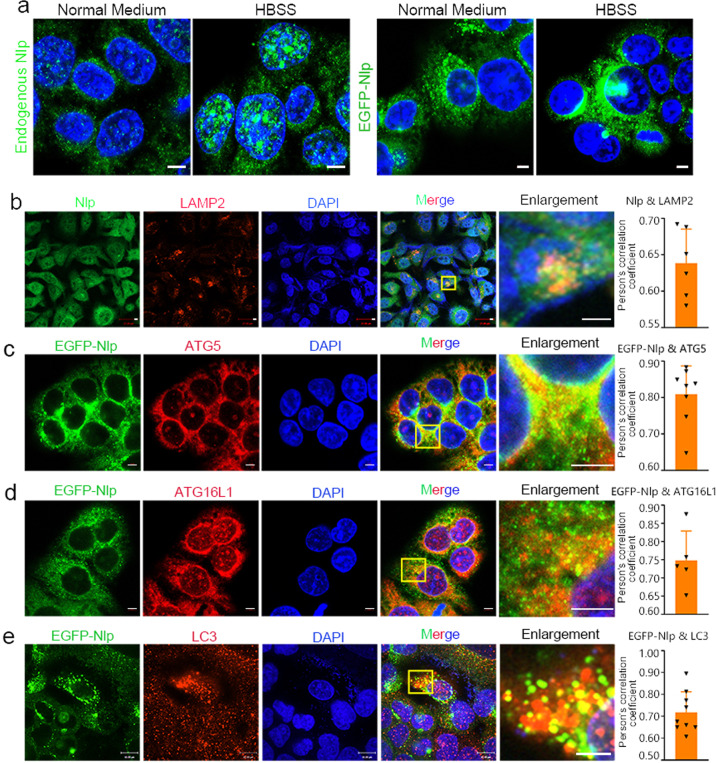
Nlp colocalized with autophagy-related proteins. **a** Hela cells and stable overexpression Hela-EGFP-Nlp cells were cultured in a complete medium or placed in HBSS for 4 h before immunofluorescence assay. The puncta of Nlp or EGFP-Nlp in the complete medium and HBSS group were compared. **b** Endogenous Nlp colocalized with LAMP2 in the cytosol of NCI-H1299 cells. **c**–**e** Overexpressed EGFP-Nlp colocalized with ATG5 (**c**), ATG16L1 (**d**), and LC3 (**e**) in Hela-EGFP-Nlp cells. The colocalization was calculated in Person’s correlation coefficient. Values are expressed as mean (bar) ± SD (error bar) for each group. (Scale bar for IF image is 5 μm)

Previous studies revealed Nlp might participate in vesicle trafficking.^30,31^ Here we sought to determine whether Nlp was implicated in autophagosome transportation. To this end, we performed a series immunofluorescence (IF) assays using laser scanning confocal microscope. We replaced the complete growth medium DMEM with Hank’s balanced salt solution (HBSS) for 4 h, which is usually used for inducing autophagy. Normal cultivated cells were used as control. We found both endogenous Nlp and overexpressed EGFP-Nlp spread in the cytosol as puncta during interphase. In the HBSS condition, the puncta of Nlp were more apparent (Fig. 1a). These observations suggested that in autophagy inducing cells, Nlp was more likely to function as puncta in the cytosol, similar to some autophagosome marker and ATG. To verify the correlation of Nlp and autophagosome, we examined the localization of Nlp (including endogenous Nlp and overexpressed EGFP-Nlp) and several autophagosome related proteins. As expected, Nlp colocalized with Beclin 1, ULK1, ATG5, ATG16L1, LC3, and LAMP2 (Figs. 1b–e and Supplementary Fig. S1a, b, c). In addition, Nlp also showed co-immunoprecipitation with several ATG, including FIP200 and Beclin 1 (Supplementary Fig. S1d). Taken together, these findings suggested that Nlp might be involved in the regulation of autophagy.

### Nlp regulates autophagy

**Fig. 2:**
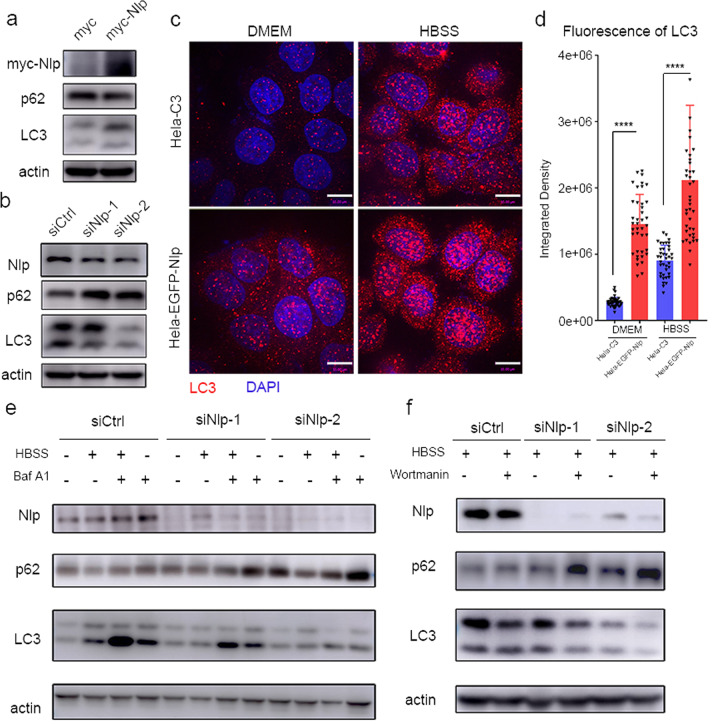
Nlp affected the number of autophagosomes and autophagic processes. **a** Western blot of cell lysates from myc-Nlp transfected Hela cells and control cells in normal medium (myc stands for myc-Nlp control vector, the same as below). Autophagy substrate adaptor p62 decreased and autophagosome marker LC3 increased. **b** Western blot of cell lysates from Nlp knockdown (siNlp) and control (siCtrl) Hela cells. LC3 decreased and p62 increased. **c** Representative images of Hela-C3 and Hela-EGFP-Nlp cells in normal medium or HBSS for 4 h. LC3 puncta (red) increased in Hela-EGFP-Nlp cells. **d** Statistics of LC3 fluorescence in (**c**). **e**, **f** Western blot of cell lysate from Hela cells under different nutrition conditions and Bafilomycin or wortmannin treatment. HBSS “-” stands for normal media DMEM, the same as below. SiNlp and siCtrl cell lysates were compared. Values are expressed as mean (bar) ± SD (error bar) for each group. (Scale bar for IF image is 10 μm. *p < 0.05, **p < 0.01, ***p < 0.001, ****p < 0.0001)

To evaluate the biological function of Nlp in autophagy, we performed a series of assays to investigate whether the expression of Nlp influenced the activity of autophagy. Immunoblotting showed that overexpression of Nlp increased the protein level of LC3 and decreased the protein level of autophagic substrate adaptor p62 in a normal medium (Fig. 2a). Meanwhile, when the expression of Nlp was knocked down by siRNA, the protein level of LC3 was decreased and p62 was increased (Fig. 2b). The alteration of LC3 and p62 indicated Nlp might promote autophagy. By IF assay, we observed that, overexpression of EGFP-Nlp induced more LC3 puncta (Fig. 2c) in both normal medium and HBSS starvation condition, and the change of LC3 fluorescence was statistically significant (Fig. 2d). In order to investigate the role of Nlp in autophagy, we detected the protein levels of LC3 and p62 in different nutrition conditions and inhibitor treatments. In the condition that fusion of autophagosome and lysosome was blocked by Bafilomycin A1, when Nlp was downregulated, the protein level of LC3 was decreased and p62 was increased both in normal medium and HBSS starvation (Fig. 2e), exhibited that the depletion of Nlp decelerated the formation of the autophagosome. And the overexpression of Nlp led to increased LC3 and decreased p62 (Supplementary Fig. S1e). When we blocked the formation of autophagosome by using Wortmannin, knockdown of Nlp induced a decrease of LC3 and increase of p62 (Fig. 2f), indicating knockdown of Nlp disrupted the fusion of autophagosome and lysosome.

**Fig. 3:**
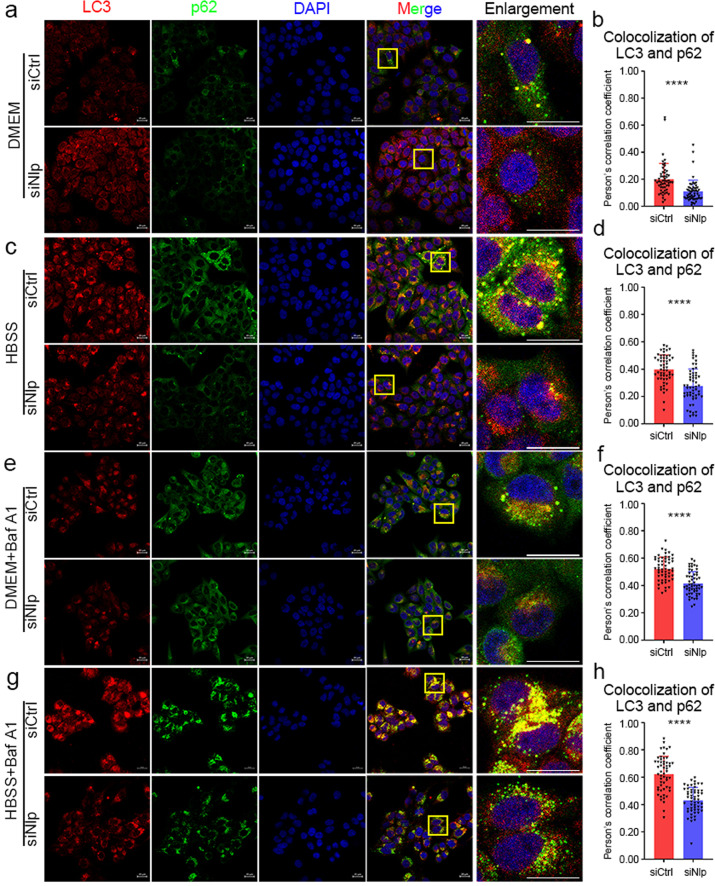
Downregulated Nlp undermined colocalization of LC3 and p62. **a**, **c**, **e**, **g** Immunofluorescence of LC3 and p62 in siNlp and siCtrl Hela cells with different nutrition conditions and Bafilomycin A1 treatment. **b**, **d**, **f**, **h** Statistics of colocalization between LC3 and p62 in (**a**), (**c**), (**e**) and (**g**), respectively. Values are expressed as mean (bar) ± SD (error bar) for each group. (Scale bar for IF image is 20 μm. *p < 0.05, **p < 0.01, ***p < 0.001, ****p < 0.0001)

**Fig. 4:**
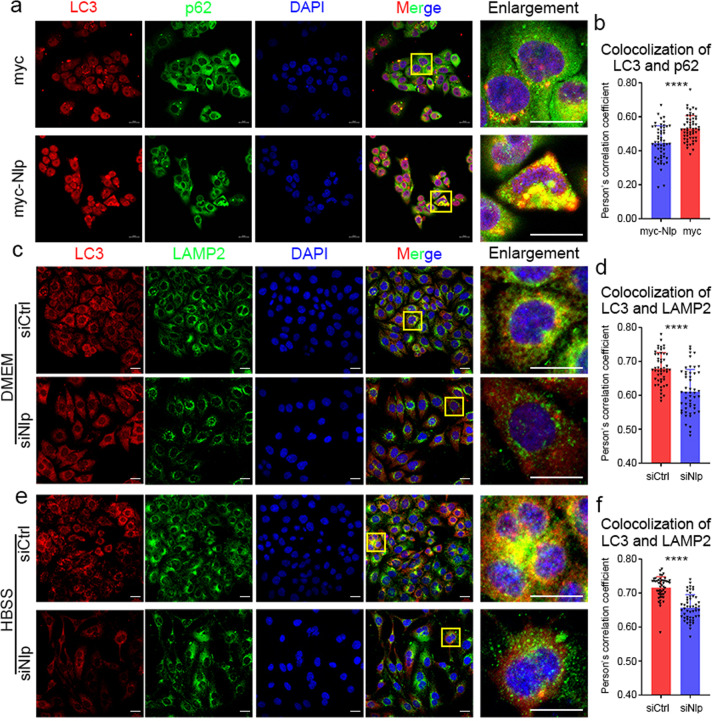
Nlp promoted colocalization between LC3 and p62, as well as LAMP2. **a** Immunofluorescence of LC3 and p62 in myc-Nlp transfected Hela cells (Nlp-overexpression) and myc-vehicle control. **b** Statistics of colocalization between LC3 and p62 in (**a**). **c**, **e** Immunofluorescence of LC3 and lysosome marker LAMP2 in siNlp and siCtrl Hela cells with different nutrition conditions. **d**, **f** Statistics of colocalization between LC3 and LAMP2 in (**c**) and (**e**), respectively. Values are expressed as mean (bar) ± SD (error bar) for each group. (Scale bar for IF image is 20 μm. *p < 0.05, **p < 0.01, ***p < 0.001, ****p < 0.0001)

Furthermore, we utilized IF assay to investigate the colocalization of the key autophagic markers, ImageJ software was employed to analyze the colocalization (technical details shown in “Materials and Methods”). In different conditions, knockdown of Nlp decreased the colocalization of LC3 and p62 (Fig. 3), meanwhile, Nlp overexpression induced the colocalization of LC3 and p62 (Fig. 4a, b). This result showed Nlp enhanced the formation of LC3 and p62. In another IF assay, we investigated the colocalization of LC3 and lysosome marker LAMP2, the results showed that colocalizations of LC3 and LAMP2 (the yellow puncta) were decreased in siNlp cells (Fig. 4c–f), which suggested Nlp might influence the fusion of autophagosome and lysosome. Collectively, Nlp might influence the pathway from the formation to fusion with the lysosome of the autophagosome.

### Nlp interacts with the key mediators of autophagosome transportation

**Fig. 5:**
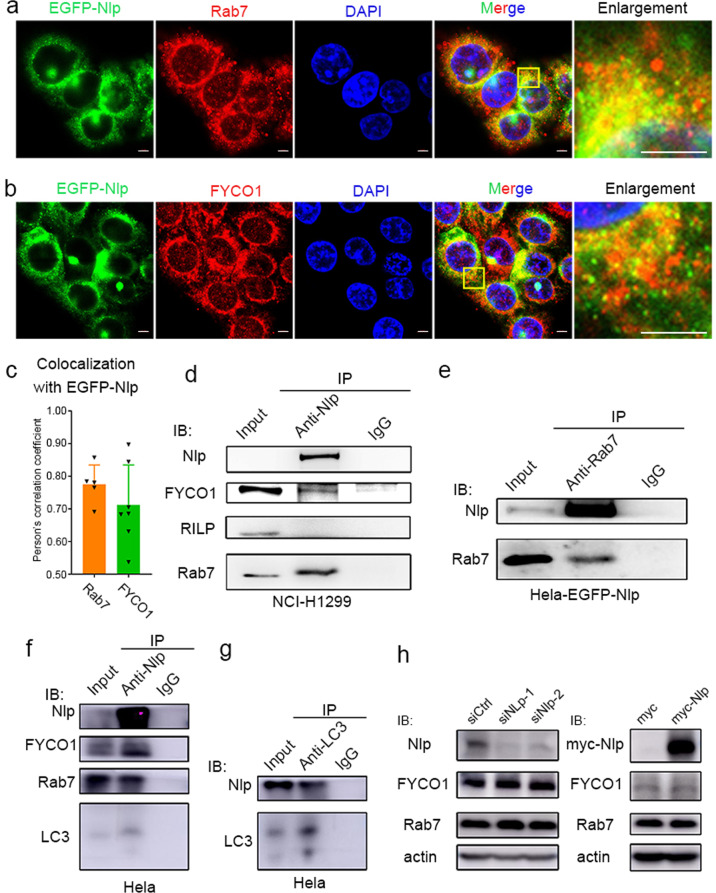
Nlp interacted with Rab7, FYCO1 and LC3. **a**, **b** EGFP-Nlp (green) colocalized with Rab7 and FYCO1 (red) in the cytosol. **c** Statistics of colocalization between EGFP-Nlp and Rab7, EGFP-Nlp and FYCO1, respectively. **d** Nlp co-immunoprecipitated with FYCO1 and Rab7, but not with RILP from NCI-H1299 cells lysate. **e** Rab7 co-immunoprecipitated with Nlp from Hela-EGFP-Nlp cells lysate. **f** Nlp co-immunoprecipitated with FYCO1, Rab7, and LC3 from Hela cells lysate. **g** LC3 co-immunoprecipitated with Nlp from Hela cells lysate. **h** The downregulation nor upregulation of Nlp didn’t affect the protein levels of FYCO1 and Rab7 in Hela cell lysate. Values are expressed as mean (bar) ± SD (error bar) for each group. (Scale bar for IF image is 5 μm)

The findings described above suggested that Nlp functioned in autophagy regulation probably through modulating the formation and fusion of autophagosome. A previous study revealed that Nlp participated in Rab8-MICAL3-regulated vesicle trafficking,^30^ we speculated that Nlp might also regulate the autophagosome transport to enhance the autophagy pathway. Since LC3 functions in autophagosome trafficking via interaction with Rab7, the key regulator of autophagosome transport,^11^ we investigated the relations of Nlp and Rab7, and its downstream effector, FYCO1. By IF assays, EGFP-Nlp was shown to colocalize with Rab7 and FYCO1, which suggested Nlp had a potential function to interact with Rab7 and FYCO1 (Figs. 5a–c and S2). Rab7, the key regulator in endo-lysosomal trafficking, is reported to govern MT minus end as well as plus end-directed vesicle transport.^16^ Rab7 binds to FYCO1 for linking Rab7 labeled autophagosome to MT plus end movement molecular motor—kinesin, while facilitates cargo in microtube minus end transportation via RILP.^19^ The co-immunoprecipitation (Co-IP) by anti-Nlp antibody confirmed Nlp interaction with Rab7 and FYCO1, as well as LC3, but not with RILP and (Figs. 5d–f and Supplementary Figs. S3a, b). In addition, Nlp was observed in the immune-complex enriched by anti-Rab7 or FYCO1 (Figs. 5e Supplementary Fig. S3c), further validating the interactions between Nlp and Rab7 or FYCO1. Consistently, Co-IP by anti-LC3 antibody also verified the interaction of LC3 and Nlp (Fig. 5g). Taken together, these results uncovered the interactions between Nlp and key mediators of autophagosome transporting, which further confirmed the role of Nlp in autophagy.

### Nlp facilitates the interaction of Rab7 and FYCO1

**Fig. 6:**
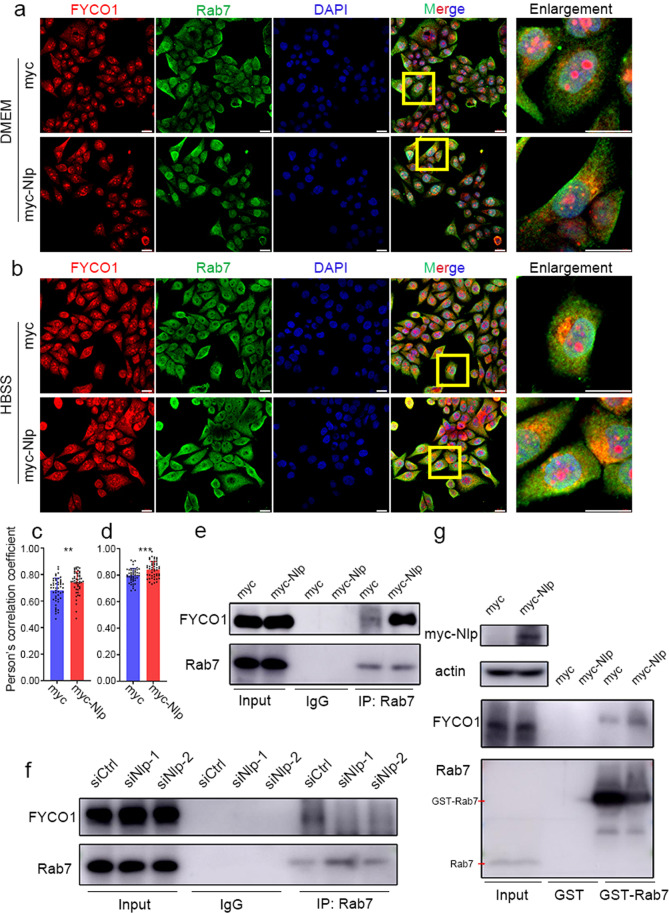
Nlp enhanced the interaction of Rab7 and FYCO1. **a**, **b** Immunofluorescence of FYCO1 and LC3 in myc-Nlp transfected Hela cells and control cells in normal medium and HBSS showed that Rab7 and FYCO1 colocalized in the cytoplasm and displayed more puncta in myc-Nlp overexpressed cells. **c**, **d** Statistic of Person’s correlation coefficient of Rab7 and FYCO1 in (**a**) and (**b**) respectively. **e** Rab7 co-immunoprecipitated more FYCO1 in myc-Nlp transfected Hela cell lysate. **f** Rab7 co-immunoprecipitated less FYCO1 in siNlp Hela cell lysate. **g** GST-Rab7 was used as an affinity matrix to incubate with FYCO1 purified from FYCO1 overexpressed Hela cell extracts, followed by Western blotting analysis with indicated antibodies to show the specific interaction. Myc-Nlp plasmid was used for detecting interaction alteration between with or without extra Nlp protein. Values are expressed as mean (bar) ± SD (error bar) for each group. (Scale bar for IF image is 20 μm. *p < 0.05, **p < 0.01, ***p < 0.001)

Given the fact that Nlp interacts with Rab7 and its downstream effector FYCO1, and Rab7 recruits FYCO1 or RILP to decide autophagosome transport to MT plus end or minus end respectively. We next sought to determine whether Nlp could affect Rab7 and FYCO1. Firstly, we evaluated whether Nlp could modulate the expression of Rab7 and FYCO1, the result showed neither overexpression nor knockdown of Nlp could change the protein levels of Rab7 or FYCO1 (Fig. 5h). This result opposed that Nlp might have an effect on the expression of Rab7 and FYCO1 directly. Rab7 functions as a regulator of cellular vesicle when it binds to downstream effector. We presumed Nlp might alter the interaction between Rab7 and FYCO1 or RILP. As shown in Fig. 6a and Supplementary Fig. S4b, IF assays confirmed the colocalization of Rab7 with FYCO1 and RILP, respectively. We utilized a cell model transient transfected with plasmid overexpressing Nlp to investigate the colocalization of FYCO1 and Rab7. We observed that the colocalization puncta of Rab7 and FYCO1 increased apparently when Nlp was overexpressed. The colocalization of Rab7 and FYCO1 was increased significantly in Nlp overexpression cells compared to control group in both normal and nutrient insufficient conditions (Fig. 6a–d), while was greatly decreased in Nlp knockdown cells in normal medium (Supplementary Fig. S4a and c). Interestingly, the colocalization of Rab7 and RILP had no significant difference between Nlp overexpression cells and the control group (Supplementary Fig. S4b, d). To further verification, we performed Co-IP assay with Rab7 antibody in lysate of Nlp overexpression and knockdown cells. Our results showed that the interaction of Rab7 and FYCO1 was enhanced in Nlp overexpression cells while attenuated in Nlp knockdown cells (Fig. 6e, f). Moreover, we performed GST pulldown assay to verify whether the direct interaction of Rab7 and FYCO1 was influenced by Nlp. GST pulldown results showed the direct interaction between Rab7 and FYCO1 was enhanced by overexpression of Nlp (Fig. 6g). Collectively, these data suggested Nlp promoted the interaction between Rab7 and FYCO1.

### Nlp promotes the formation of autophagolysosome

**Fig. 7:**
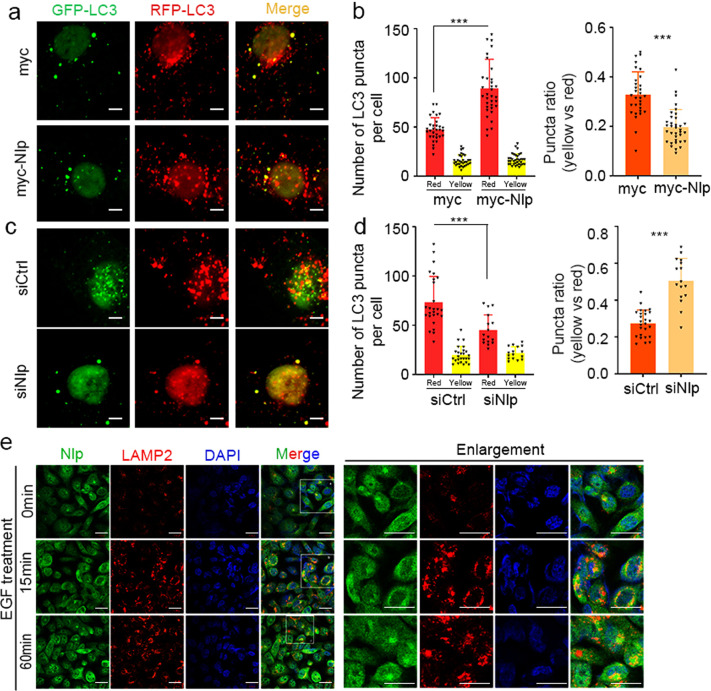
Nlp facilitated fusion of autophagosome and lysosome. **a**, **c** Myc-Nlp plasmid and siRNA of Nlp was transfected into Hela cells transient transfected with RFP-GFP-LC3 plasmid for 48 h and cells media were subsequently replaced by HBSS for another 8 h before IF assay. Representative images of fluorescent LC3 puncta were shown. **b**, **d** Statistic of LC3 puncta number and puncta ratio (yellow vs red). **e** EGF was added to the medium for a different time before IF assay, representative images showed the colocalization of Nlp and LAMP2 increased after EGF treatment in NCI-H1299 cells. Values are expressed as mean (bar) ± SD (error bar) for each group. (Scale bar for IF image is 5 μm. *p < 0.05, **p < 0.01, ***p < 0.001)

**Fig. 8:**
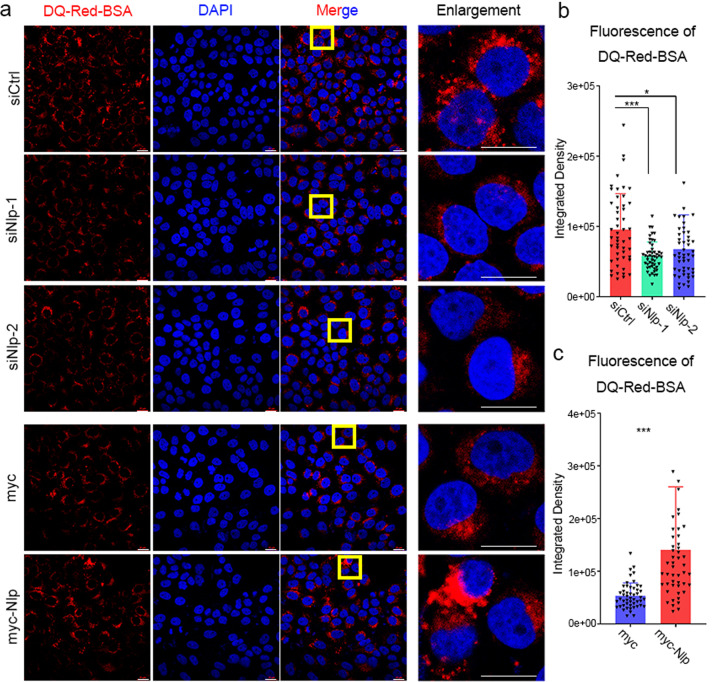
Nlp promote DQ-Red-BSA induced lysosomal transport. **a** Representative immunofluorescence images of Nlp knockdown and overexpressed Hela cells and control cells. **b** Statistics of red fluorescence density in each group. Values are expressed as mean (bar) ± SD (error bar) for each group. (Scale bar for IF image is 20 μm. *p < 0.05, **p < 0.01, ***p < 0.001)

FYCO1 has been reported to link autophagosome to MT plus end movement motor kinesin, which promotes maturation of autophagosome and formation of autophagolysosome.^32^ Thus, we hypothesized that Nlp might affect the formation of autophagolysosome via Rab7/FYCO1. To probe this notion, we monitored autophagy flux using the mRFP-GFP-LC3 reporter system. It should be noted here that GFP is sensitive to an acidic environment, when autophagosome fuses with a lysosome, GFP protein will quickly denature and green fluorescence is quenched, and only red fluorescence of RFP can be detected. Thus, if GFP and RFP can be detected in the same puncta simultaneously, it indicates that autophagosome has not been fused with lysosome. When Nlp was upregulated, the numbers of red puncta substantially increased, and the yellow-to-red puncta ratio decreased (Fig. 7a, b). On the contrary, the numbers of red puncta decreased, and the yellow-to-red puncta ratio increased while Nlp was knockdown (Fig. 7c, d). The results indicated that Nlp promoted autophagosome transport to the lysosome in autophagy. Nlp might have a potential function in lysosome-related vesicle transport. We also examined the colocalization of Nlp and lysosome in another condition, when cells were treated with EGF to induce the endosome transport, Nlp was found to colocalize with LAMP2 (Fig. 7e). These evidences indicated that Nlp may be involved in the regulation of lysosome. For further confirmation of the function of Nlp in intracellular transport to the lysosome, we incubated cells with DQ-Red-BSA, an endocytic cargo engulfed by cells via endocytosis that becomes fluorescent upon proteolytic cleavage in lysosomes. The level of DQ-Red-BSA fluorescence reflects the activity of endosome and autophagosome transport to the lysosome. Results showed that red fluorescence intensified in Nlp overexpressed cells and weakened in siNlp cells, indicating that Nlp promoted endocytic cargo transport to lysosome (Fig. 8). Nlp exhibited function in promoting autophagic degradative transport. Together, these observations demonstrated that the formation of autophagolysosome was regulated by Nlp.

### NLP deficient mice are prone to suffering hepatomegaly and liver cancer

**Fig. 9:**
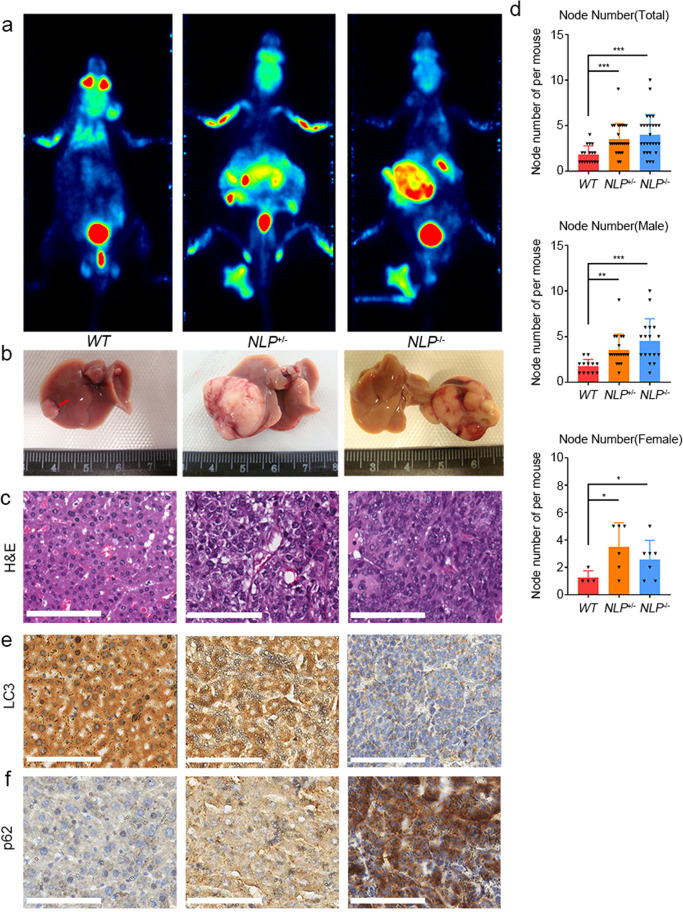
Nlp deficient mice had higher incidence of hepatomegaly and liver cancer. **a** Representative PET images of DMBA-induced hepatoma of *WT*, *NLP*^+/−^, and *NLP*^−/−^ mice. **b** Photos of the liver with tumor dissected from mice in (**a**). **c** H&E of the tumor of mice in (**b**) respectively. **c** IHC images of LC3 images of the tumors in (**b**) respectively. **e** IHC images of p62 of the tumors in (**b**) respectively. **f** Statistic of the liver weight of DMBA treated *WT*, *NLP*^+/−^, and *NLP*^−/−^ mice. **d** Statistic of node number of DMBA treated *WT*, *NLP*^+/−^, and *NLP*^−/-^ mice (total, male and female, respectively). Values are expressed as mean (bar) ± SD (error bar) for each group. (Scale bar for H&E and IHC image is 100 μm. *p < 0.05, **p < 0.01, ***p < 0.001)

Autophagy was reported as an inhibition of hepatomegaly^33^ and DMBA/TPA-induced liver cancer.^34^ We have recently generated a Nlp knockout mice model to study the biological functions of Nlp (Supplementary Fig. S5a, b). To investigate the effect of *NLP* dysfunction in an animal model, we treated *NLP* deficient mice with one polycyclic aromatic hydrocarbon (PAH)—7,12-dimethyl benzanthracene (DMBA) and recorded phenotypic changes. *NLP* deficient mice grew up as normal. During the experiment, we performed a positron emission tomography (PET) scan to examine the tumorigenesis in vivo. PET images indicated tumor incidence in liver area, and the tumor in *NLP*^+/−^ and *NLP*^−/−^ mice were more apparent (Fig. 9a), suggesting that DMBA was able to induce liver cancer in mice, especially *NLP*^+/−^ and *NLP*^−/−^ mice. At the endpoint (14 months), we euthanatized all the mice and examined the tumor development. Consistent with the PET results, the mice developed liver cancer (Fig. 9b, c). The DMBA treated mice developed a variety of tumors, mainly liver cancer, while other cancers, including lymphoma, colon cancer, and lung cancer, displayed a relatively low incidence (data not shown). The liver cancer incidence of each genotype and gender is shown in Table 1. While in the control group (mice were not treated with DMBA), there was only one liver cancer case in *NLP*^+/−^ and *NLP*^−/−^ mice, respectively (Supplementary Table S1).

**Table 1:** Hepatoma incidence in DMBA-induced *NLP* defect and *WT* mice

DMBA	Total			Male			Female		
	*WT*	*NLP*^+/−^	*NLP*^−/−^	*WT*	*NLP*^+/−^	*NLP*^−/−^	*WT*	*NLP*^+/−^	*NLP*^−/−^
No. of mice	48	49	44	25	25	23	24	24	21
Hepatoma	15	24	24	12	18	16	3	6	8
Incidence (%)	31.25%	48.98%	54.55%	48.00%	72.00%	69.57%	12.50%	25.00%	38.10%

Firstly, we observed that the incidence of liver cancer in all the DMBA groups was relatively high, and the incidence of liver cancer in wild-type (*WT*), *NLP*^+/−^ and *NLP*^−/−^ mice were 31.25% (15/48), 48.98% (24/49) and 54.55% (24/44), respectively. According to the statistical analysis of Fisher’s exact test, the incidence of liver cancer in the genetically defective mice of *NLP*^+/−^ and *NLP*^−/−^ groups was significantly higher than that of the *WT* mice, and the differences were statistically significant (*p* value between the *NLP*^+/−^ groups and *WT* groups was 0.048, *NLP*^−/−^ groups and the *WT* groups was 0.02, respectively). By Chi-square test, in male mice, the incidence of liver cancer in the genetically deficient mice (*NLP*^+/−^ (16/23) and *NLP*^−/−^ (18/25) groups) was higher than that in the *WT* groups (12/20), but the differences were not statistically significant (all *p* values were >0.05). In female mice, there was no statistically significant difference in the incidence of liver cancer between the *NLP*^+/−^ defect group (6/24) and the *WT* (3/24) mice after the corrected Chi-square test, and there was a statistically significant difference in the incidence of liver cancer between the *NLP*^−/−^ defect group (8/21) and the *WT* mice after the Chi-square test (*p* = 0.046). We measured the collected liver cancer specimens in DMBA group and found that the number of nodules in the *NLP*^+/−^ and *NLP*^−/−^ male and female mice were significantly higher than that seen in the *WT* animals (Fig. 9d). To identify the effect of *NLP* deficient on hepatomegaly, we compared the tumor-free liver (without liver tumor and liver metastasis) of each genotype in weight and liver-to-body ratio (liver ratio), found that mean value of liver weight and liver ratios of *NLP*^+/−^ and *NLP*^−/−^ mice were significantly greater than the *WT* mice. The difference of female liver weight between *NLP*^+/−^ and *WT* mice had statistical significance (Fig. S6a). In addition, the liver ratio of *NLP*^−/−^ mice was significantly greater than *WT* mice, and liver ratio of *NLP*^+/−^ female mice was significantly greater than *WT* female mice (Fig. S6b).

Because autophagy defect leads to hepatoma and hepatomegaly, we wondered whether autophagy was impaired in the liver and tumor. We performed IHC assay to detect the autophagy marker LC3 and p62 in liver tumor and tumor-free liver tissue of *WT*, *NLP*^+/−^ and *NLP*^−/−^ mice, found that in *NLP*^+/−^ and *NLP*^−/−^ mice, LC3 was downregulated while p62 was upregulated, in both tumor and liver. (Figs. 9e, f and Supplementary Fig. S5e, f). The IHC results indicated that dysfunction of autophagy might be one of the major reasons for increased liver cancer incidence and liver weight. Taken together, deficiency of Nlp in mice increased hepatomegaly and tumorigenesis, which might be partially resulted from impaired autophagy of liver cell.

## Discussion

During autophagy, cellular vesicle cargo including autophagosome and lysosome are driven by molecular motor along the MT.^35^ This process is orderly and precisely regulated by a series of proteins. Among them, Rab7, which is defined as the main sorting protein of the endosome and autophagosome transport network, is responsible for the determinant of transport directions.^36^ The binding of Rab7 and its downstream effector FYCO1 promotes the maturation and MT plus end transport of autophagosome. In this study, we demonstrated that centrosome associated protein Nlp was a novel autophagosome regulator. Nlp was colocalized with various ATG including Beclin 1, ULK1, ATG5, ATG16L1, LC3, and LAMP2. The co-immunoprecipitation assay showed that Nlp was able to interact with LC3. As a key regulator of vesicle transport, Rab7 and its downstream effector FYCO1 play essential roles of autophagosome sorting and maturation. FYCO1 that binds to Rab7 is responsible for the recruitment of kinesin, the molecular motor for autophagosome movement toward plus end of MT. This process is followed by the fusion of autophagosome and lysosome. Interestingly, we found that Nlp enhanced the binding of FYCO1 to Rab7. The colocalization of FYCO1 and Rab7 was upregulated upon Nlp overexpression. When Nlp was depleted, the colocalization of FYCO1 and Rab7 was attenuated. Co-immunoprecipitation assay also confirmed the effect of Nlp expression on the interaction of FYCO1 and Rab7. Importantly, Nlp could promote the formation of autophagolysosome. These findings have provided a new mechanism by which centrosome related protein Nlp regulates autophagy via Rab7/FYCO1 and extended our understanding on autophagosome transportation.

Ninein-like protein Nlp was first reported as a regulator of mitosis. But its dysfunction also induces disruption in the vesicle system. The previous finding has linked Nlp to traffic observed dispersion of Golgi and lysosome following Nlp overexpression.^29^ In zebrafish, loss of ninein results in defects in brain and skull development of zebrafish, while co-function of Nlp with DZANK1 in vesicle transport is essential for photoreceptor development.^31^ Nlp also associates with ciliopathy protein CC2D2A and functions in Rab8-MICAL3-regulated vesicle trafficking.^30^ Through a BioID mass spectrometry screen for interactome of dynein–dynactin, which is responsible for MT minus end trafficking of cargo, Nlp has been identified as an activating adaptor of dynein.^37^ Since we have observed that Nlp displays bi-directional movement along MT in the previous experiments, it was speculated that Nlp might also participate in another process of vesicle transport, such as autophagy. Ninein and ninein like proteins have not yet been reported to involve in the cargo regulation of autophagosome. Here, we provided the first demonstration that the Nlp plays role in autophagosome transport.

FYCO1 has been reported as a Rab7 effector and could link kinesin to Rab7 regulated endosome and autophagosome.^18^ Like dynein, kinesin superfamily proteins (KIFs) are also important molecular motors that directionally transport cargos along MT. One type of kinesins—N-kinesin group is responsible for MT plus end transport.^38^ In the beginning of autophagy, FYCO1 binds to N-kinesin and redistributes pre-autophagosome membrane and interact with Rab7 and LC3, then link autophagosome to this molecular motor.^20^ The long coiled-coil region of FYCO1 is considered as the binding motif for kinesin.^18^ But N-kinesin group has different proteins, how FYCO1 recognizes the right kinesin is unrevealed. Nlp has been reported to bind dynein in order to achieve centrosome (minus end terminal of MT) to function in mitosis,^25^ while overexpression of Nlp leads to dispersion toward cell periphery of lysosome in interphase.^29^ As some dynein adaptors have been suggested to act as bidirectional adaptors since they can interact directly with both dynein and kinesin.^39^ In many cases, the region for dynein and kinesin interaction overlaps, suggesting they may switch in bidirectional transport. It’s reported that Kif3a—a kinesin II subunit, facilitates the formation of centriole via interacting with dynactin subunit p150^Glued^ and ninein.^40^ Nlp contains four coiled-coil domains similar to other motor activator and EF-hand domains similar to Rab protein.^30^ These domains might be responsible for interaction with FYCO1-kinesin and Rab7 on the autophagosome membrane respectively. It is reasonable to speculate that Nlp may act as an activator for Rab7-FYCO1-kinesin complex. Once Nlp-Rab7-FYCO1 complex is formed, autophagosomes would be linked to kinesin. Then autophagosomes can be driven by kinesin along the MT to plus end and to distribute in the cell periphery. Therefore, it is worthy to evaluate this process in promoting the maturation of autophagosome and the formation of autophagolysosome.

Defective autophagy in mice leads to hepatomegaly, liver injury and spontaneous liver tumorigenesis. Liver-specific deletion of ATG5 and ATG17 mice display hepatic metabolic dysfunction which results in liver injury, hepatomegaly, inflammation, fibrosis and benign tumors.^33,41^ When autophagy is impaired, p62 accumulates and binds to KEAP1, which negatively regulates and stabilizes NRF2, that leads to a deleteriously high antioxidative response accelerating liver injury and metabolic reprogramming.^42^ This phenotype can be attenuated by deletion of Yap or p62.^43^ Autophagy has been known to play a dual role in liver cancer.^44^ In advanced or metastatic tumor cells, autophagy may act as a maintaining process to promote the survival of tumor cells in low-nutrient condition and chemotherapy stress.^45^ However, during the initiation of cancer, autophagy maintains cell homeostasis through clearance of damaged organelles and reactive oxygen species, which inhibits malignant transformation and tumorigenesis.^44^ Several proteins in autophagy pathway have been reported as suppressors of hepatocarcinogenesis. In addition to ATG5 and ATG17, autophagic protein Beclin 1 deficient mice model has a high incidence of hepatocellular carcinoma.^46^ Endocrines such as thyroid hormone suppresses hepatocarcinogenesis in diethylnitrosamine (DEN) induced mice model, indicating autophagy may be a gatekeeper in carcinogen-induced liver cancer.^47^ In other chemical carcinogen-induced tumorigenesis, autophagy plays a protective mechanism that inhibits DMBA/TPA-induced skin cancer.^34^ All the evidences indicate that autophagy-related genes could be a promising suppressor of spontaneous and induced tumorigenesis. In accord with these previous reports, our study revealed that Nlp played a key role in the regulation of autophagy, and *NLP* deficient mice displayed increased tumor weight, tumor-body weight ratio and tumor incidence in DMBA inducement.

In summary, this study has demonstrated that Nlp was involved in autophagy and promoted the formation of autophagolysosome through facilitating the interaction between Rab7 and FYCO1, which was implicated with tumorigenesis of liver cancer.

## Materials and methods

### Antibody

The following antibodies were used: anti-LAMP2 rabbit polyclonal antibody (Abcam); anti-LAMP2 mouse monoclonal antibody (Abcam); anti-Rab7 mouse monoclonal antibody (Abcam); anti-Rab7 rabbit polyclonal antibody (Proteintech); anti-FYCO1 rabbit polyclonal antibody (Proteintech); anti-RILP rabbit polyclonal antibody (Proteintech); anti-p62/SQSTM1 rabbit polyclonal antibody (Proteintech); anti-p62/SQSTM1 mouse monoclonal antibody (Abcam); anti-LC3 rabbit polyclonal antibody (Proteintech); anti-LC3 rabbit polyclonal antibody (CST); anti-LAMP2 mouse monoclonal antibody (Proteintech); anti-ATG5 mouse monoclonal antibody (Proteintech); anti-ATG16L1 rabbit polyclonal antibody (Proteintech); anti-Beclin 1 rabbit polyclonal antibody (CST); anti-ULK1 rabbit monoclonal antibody (CST); anti-FIP200 rabbit monoclonal antibody (CST); anti-NINL rabbit polyclonal antibody (Sigma-Aldrich); anti-Nlp rabbit polyclonal antibody (MBL BEIJING BIOTECH); anti-β-actin mouse monoclonal antibody (Sigma-Aldrich); HRP-conjugated AffiniPure Mouse Anti-Rabbit IgG Light Chain (ABclonal); Alexa Fluor® 647-conjugated rabbit-anti-mouse IgG (Thermo Fisher); TRITC-conjugated rabbit-anti-mouse IgG (Thermo Fisher); FITC-conjugated goat-anti-rabbit and goat-anti-mouse IgG (ZSGB-BIO); TRITC-conjugated goat-anti-rabbit and goat-anti-mouse IgG (ZSGB-BIO); Alexa Fluor® 488-conjugated goat-anti-mouse IgG (ZSGB-BIO); Alexa Fluor® 594-conjugated goat-anti-mouse IgG (ZSGB-BIO); FITC-conjugated rabbit-anti-goat IgG (ZSGB-BIO).

### Animals

*NLP* deficient mice were generated by Institute of Laboratory Animals Science, CAMS & PUMC. All mice were kept at 22 ± 2 °C on 12-h light-dark cycles with ad libitum access to food and water. All animal care and procedures were in accordance with national and institutional policies for animal health and well-being. Mice were used according to the protocols approved by Institutional Animal Care and Use Committee of Cancer Institute and Hospital, Chinese Academy of Medical Sciences and Peking Union Medical College (Beijing, China).

### Cell culture and transfection

Stable Hela-EGFP-Nlp and control Hela-C3 cells were generated by our laboratory previously.^27^ All cells were cultured at 37 °C in a 5% CO_2_ atmosphere. Hela and Hela derived cell lines, HEK293T and ZR-75-1 were cultured with DMEM medium supplemented with 10% heat-inactivated fetal bovine serum (FBS) and 1% penicillin/streptomycin. NCI-H1299 were cultured with PRIM-1640 medium supplemented with 10% heat-inactivated FBS and 1% penicillin/streptomycin.

All transient transfection, including siRNA knocking down and overexpression, was performed using Lipofectamine reagent according to manufacturer’s instruction (Invitrogen). Cells were harvested 24–96 h post transfection.

### Immunoblotting

Cell protein lysates were prepared using 1× PBS supplemented with 1% Nonidet P-40, 1× protease inhibitors cocktail (Roche) and 50 mg/ml phenylmethylsulphonyl fluoride. Proteins were resolved by SDS–PAGE and transferred to polyvinylidene difluoride membrane. Membranes were incubated with the indicated primary antibodies and anti-mouse or anti-rabbit secondary antibodies conjugated with horseradish peroxidase (HRP). After addition of HRP substrate, the chemiluminescence signal was detected with Luminescent Image Analyzer LAS-4000 (Fujifilm).

### Co-Immunoprecipitation and GST pull-down assay

For co-immunoprecipitation, cells were lysed with lysis buffer (20 mM Tris/HCl, pH = 7.6, 100 mM NaCl, 20 mM KCl, 1.5 mM MgCl_2_, 0.5% Nonidet P-40, 1× protease inhibitors cocktail (Roche)). Protein A/G Magnetic Beads (MCE) were incubated with ~2 μg antibody or IgG at 4 °C for 1 h, then incubated with cell lysates at 4 °C overnight. The precipitates were washed with lysis buffer for five times before preparation for immunoblotting assay.

For GST pull-down, GST-Rab7 fusion protein was purified by Glutathione High Capacity Magnetic Agarose Beads (Sigma). After Glutathione Magnetic Agarose Beads—conjugated GST fusion proteins were mixed with cell lysates (3 mg) for 6 h at 4 °C, it was washed five to seven times with lysis buffer and boiled in loading buffer. The binding proteins were analyzed by Western blotting assay.

### Confocal microscopy analyses

Cells were seeded on coverglass slides in confocal dishes (Nest Scientific USA Inc.). After 12 h or overnight, medium was removed. Cells were washed gently with 1×PBS, then fresh complete medium or HBSS was added to each well respectively. After 4 h incubation, cells were washed by 1×PBS twice before fixation with 4% paraformaldehyde for 10 min. Then cells were washed with 1×PBS and treated by 0.1 M glycine solution shortly, and permeabilized with 0.4% TritonX-100 for 10 min, and blocked with 5% serum in PBS for 30 min. Subsequently, cells were incubated with primary antibodies at 4 °C overnight then secondary antibodies at room temperature for 1 h. Since EGFP-Nlp overexpressed and control Hela cells expressed EGFP, we employed Alexa Fluor® 647 (purple) -conjugated IgG as the fluorescent secondary antibody to stain Rab7, and TRITC (red)-conjugated IgG for FYCO1 and RILP. During image analysis, we changed purple fluorescence to green for better displaying. Image were collected using a laser-scanning confocal microscope system (Leica Microsystems Heiderg GmbH, Am Friedensplatz 3, Germany).

### DQ-Red-BSA trafficking assay

Cells were seeded in confocal dishes before replacing culture medium (DMEM + 1%FBS + 1%NEAA + 1%GlutaMax + 1%HEPES) to DQ-Red-BSA (Invitrogen) at a working concentration of 10 µg/ml in culture medium for 6 h at 37 °C and 5% CO_2._ The cells were fixed in 4% PFA in PBS (pH 7.4) and analyzed using a confocal microscope.^48^ Total fluorescence intensity of DQ-Red-BSA were quantified using ImageJ software.

### Autophagic flux analysis

Cells used in autophagic flux analysis were transient transfection with mRFP-GFP-LC3 fusion protein plasmid with Nlp expressing plasmid and its control plasmid or Nlp siRNA via Lipofectamine for 48 h. Then cells were incubated with HBSS for 8 h before fixation. The number of RFP and GFP puncta were counted and statistically analyzed.

### Autophagy inhibitor treatment

Cells were seeded in dishes before inhibitor treatment. Wortmannin (Selleck, S2758) was added to a working concentration of 100 nM in normal medium for 2 h at 37 °C and 5% CO_2_. Bafilomycin A1 (Sigma, 19–148) was added to a working concentration of 10 nM for 1 h. After treatment, cells were harvested for WB assay or fixed for IF assay.

### Immunofluorescence and colocalization analysis

ImageJ (Image Processing and Analysis in Java, National Institutes of Health) was used for immunofluorescence and colocalization analysis. We used the *JACoP (Just Another Colocalization Plugin)* plugin (developed by Fabrice P. Cordelieres, Institut Curie, Orsay (France). Fabrice. Cordelieres at curie.u-psud.fr Susanne Bolte, IFR 83, Paris (France). Susanne Bolte at upmc.fr). Images of each single cell was cropped randomly and the colocalization index was analyzed individually. The colocalization quantitative index is Pearson’s correlation coefficient (range from −1.0 to +1.0, The result is +1.0 for perfect correlation, 0 for no correlation, and −1.0 for perfect anti-correlation).^49^ For a two-sample *t*-test, at least 50 cells per group were calculated in each experiment.

### Mice genotyping and 7,12-dimethyl benzanthracene (DMBA) treatment

The tail tips of new born mice were cut for genotyping in the 10^th^ day after birth. The tail tips were used for DNA extraction by using the TIANamp Genomic DNA Kit (TIANGEN). Two micrograms of genome DNA was used as template for PCR. The sequences of primers are list below:

m-N-1-F: CTCCCCTCCCCATTTTCTTAC

m-N-2-R: CAGTGGTCCAGGCTCTAGTTTTG

m-N-3-F: GAGGGTTTATTGGATACACGG

m-N-4-R: AACAGCACCTTTGATGCCTAC

m-N-1a-F: TGGGATCTGATTCCCTCTTCT

The primer pairs and PCR products were described in Fig. S5a and S5b.

*WT*, *NLP*^+/−^ and *NLP*^−/−^ mice (6–7 g in weight) were administrated with DMBA solution in the 14th day after birth. DMBA (4 mM) was diluted in maize oil and intraperitoneal injected to mice in a dose of 5 μl per gram of weight. After injection, mice were fed with normal diet after separation from mother in the 21st day.

### PET assay

Before PET imaging, the mice were fasted for 12 h and then ~200 ± 10 μCi 18-fluoro-6-deoxy-glucose (FDG) was injected by intraperitoneal injection. After 60 min of FDG uptake, mice were anesthetized with 2% isoflurane. Images were obtained with the static scanning pattern (10 min) by the Trans-PET BioCaliburn 700 system (Raycan Technology Co., Ltd, Suzhou, China). The PET images were reconstructed using the three-dimensional (3D) OSEM method with a voxel size of 0.5 × 0.5 × 0.5 mm^3^. A volume-of-interest (VOI) analysis was conducted using the AMIDE software package (The Free Software Foundation Inc., Boston, Massachusetts, USA). The mean standardized uptake value (SUV) was calculated using the following formula: mean pixel value with the decay-corrected region-of-interest activity (μCi/kg)/(injected dose [μCi]/weight [kg]).

### Immunohistochemistry

Immunohistochemistry (IHC) assay was performed by paraffin-embedded tumor and liver tissue. Before staining, the sections were deparaffinized with xylenes and rehydrated by graded ethanol. Then sections were submerged into EDTA antigenic retrieval buffer and microwaved for slightly boiling to retrieve antigen. After treatment with 3% hydrogen peroxide to quench endogenous peroxidase activity, sections were incubated with 1% goat serum albumin to block nonspecific binding. Next, Sections were incubated with diluted rabbit anti-LC3 or p62/SQSTM1 primary antibody (1:200) in 4 °C overnight. After washing in PBS, sections were treated with goat anti-mouse/rabbit IgG HRP-polymer (ZSGB-BIO, Beijing, China) for 20 min, 3, 3′-diaminobenzidine was used for color developing.

### Statistical analysis

Statistical analysis was conducted with GraphPad Prism (version 8.0 for Windows, GraphPad Software, San Diego California USA, www.graphpad.com) and R software (version 4.0.2; http://www.Rproject.org). The reported statistical significance levels were all two-sided, with statistical significance set at 0.05.

## Supplementary information

Supplemental Material

## Data Availability

The data sets that support the findings of this study are available in this paper or the Supplementary Information.
