# Supplementary material for: Nlp promotes autophagy through facilitating the interaction of Rab7 and FYCO1
Source: Signal Transduct Target Ther. 2021 Apr 16;6:152. doi: 10.1038/s41392-021-00543-1 (PMC8050283; doi:10.1038/s41392-021-00543-1)
Supplement: Supplementary file 1 — Supplemental Material [file 41392_2021_543_MOESM1_ESM.docx]

Supplementary materials for

Nlp promotes autophagy through facilitating the interaction of Rab7 and FYCO1

Wenchang Xiao^1,3^, Danna Yeerken^2^, Jia Li^1^, Zhangfu Li^4^, Lanfang Jiang^1^, Dan Li^1^, Ming Fu^1^, Liying Ma^1^, Yongmei Song^1^, Weimin Zhang^2,5*^, Qimin Zhan^1,2,5,6*^

^*^Correspondence to Qimin Zhan ([zhanqimin@bjmu.edu.cn](mailto:zhanqimin@bjmu.edu.cn)) Tel.: (+86) 010-88196614 or Weimin Zhang ([wmzhang411@163.com](mailto:wmzhang411@163.com)) Tel.: (+86) 010-88196375.

**This file includes:**

Table S1

Figures S1 to S6

**Table S1. Tumorigenesis in control *NLP* deficient and *WT* mice.**

| **NC** | *WT* | *NLP*^+/-^ | *NLP*^-/-^ |
| --- | --- | --- | --- |
| No. of mice | 32 | 34 | 27 |
| Hepatoma | 0 | 1 | 1 |
| Incidence (%) | 0% | 2.94% | 3.70% |


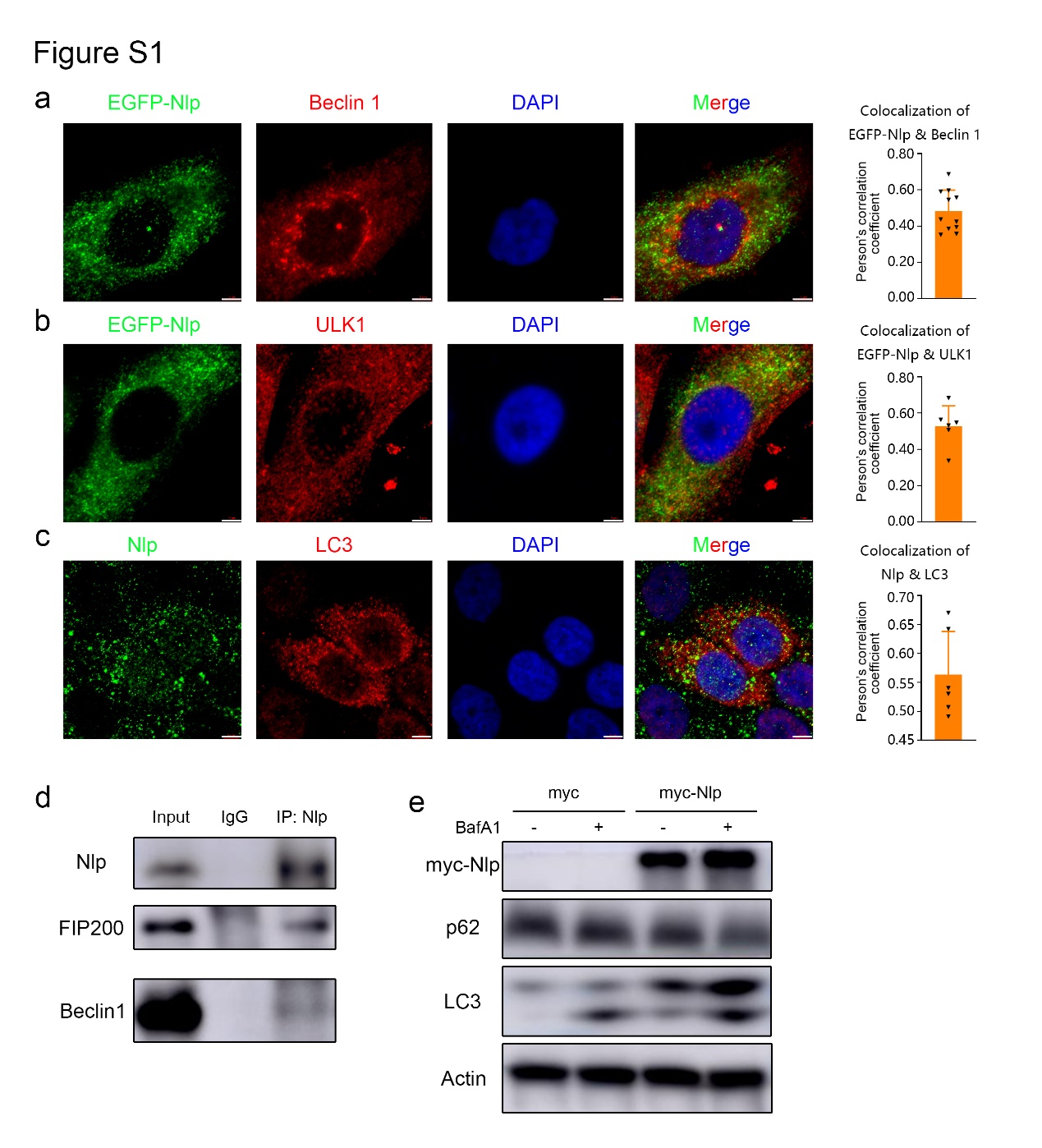


**Figure S1.** (a, b) Nlp (green) colocalized with Beclin 1, ULK1 in Hela-EGFP-Nlp cells and (c) LC3 in Hela cells. (d) Nlp coimmunoprecipitated with FIP200 and Beclin 1. (e) Overexpression of Nlp increased LC3 and decreased p62. Values are expressed as mean (bar) ± SD (error bar) for each group. (Scale bar for IF image is 5 μm.)


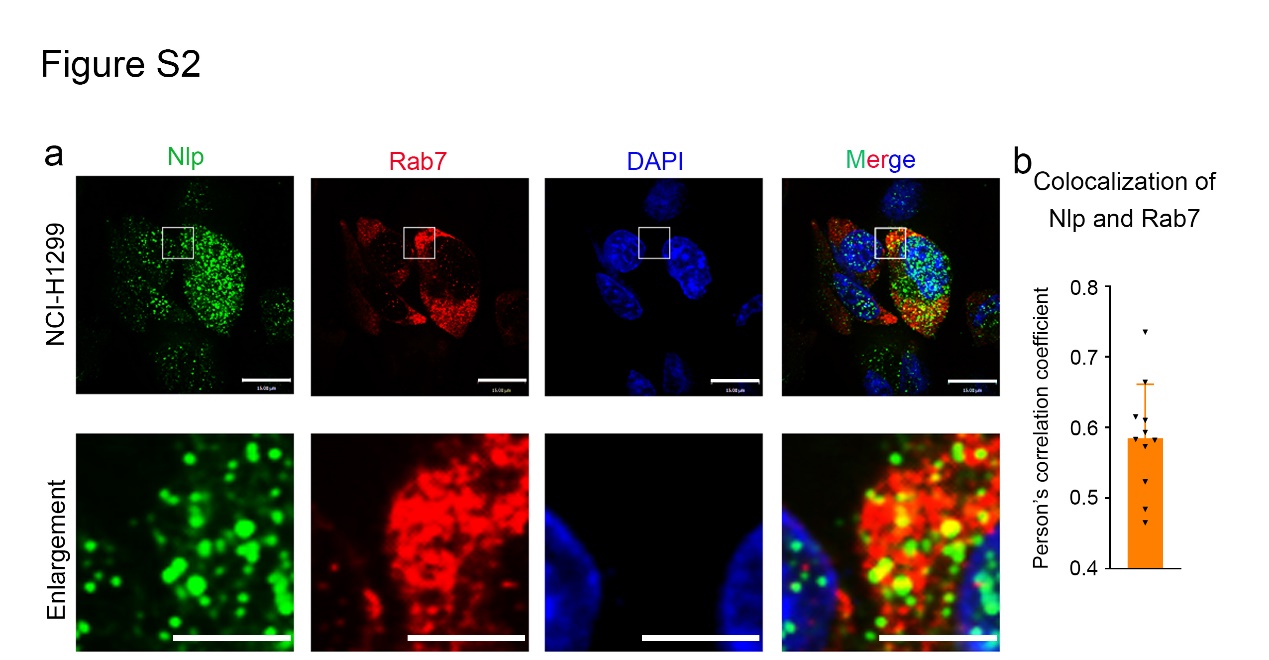


**Figure S2.** Endogenous Nlp (green) and Rab7 (red) colocalized in cytosol. Values are expressed as mean (bar) ± SD (error bar). (Scale bar for IF image is 10 μm.)


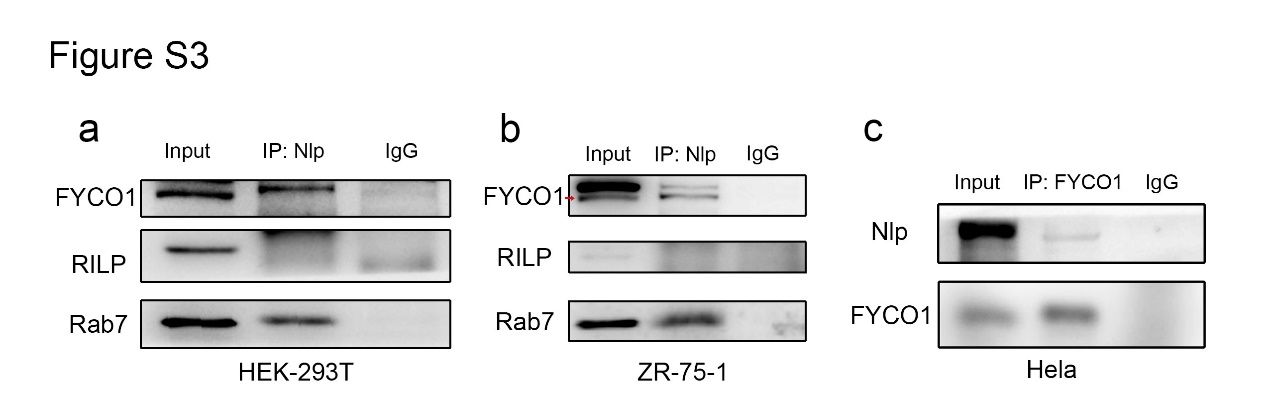


**Figure S3. Nlp interacted with Rab7 and FYCO1.** (a) Nlp coimmunoprecipitated with FYCO1 and Rab7, but not with RILP from HEK-293T cells lysate. (b) Nlp coimmunoprecipitated with FYCO1 and Rab7, but not with RILP from ZR-75-1 cells lysate. (c) Nlp was existed in the FYCO1 enriched immunoprecipitates.


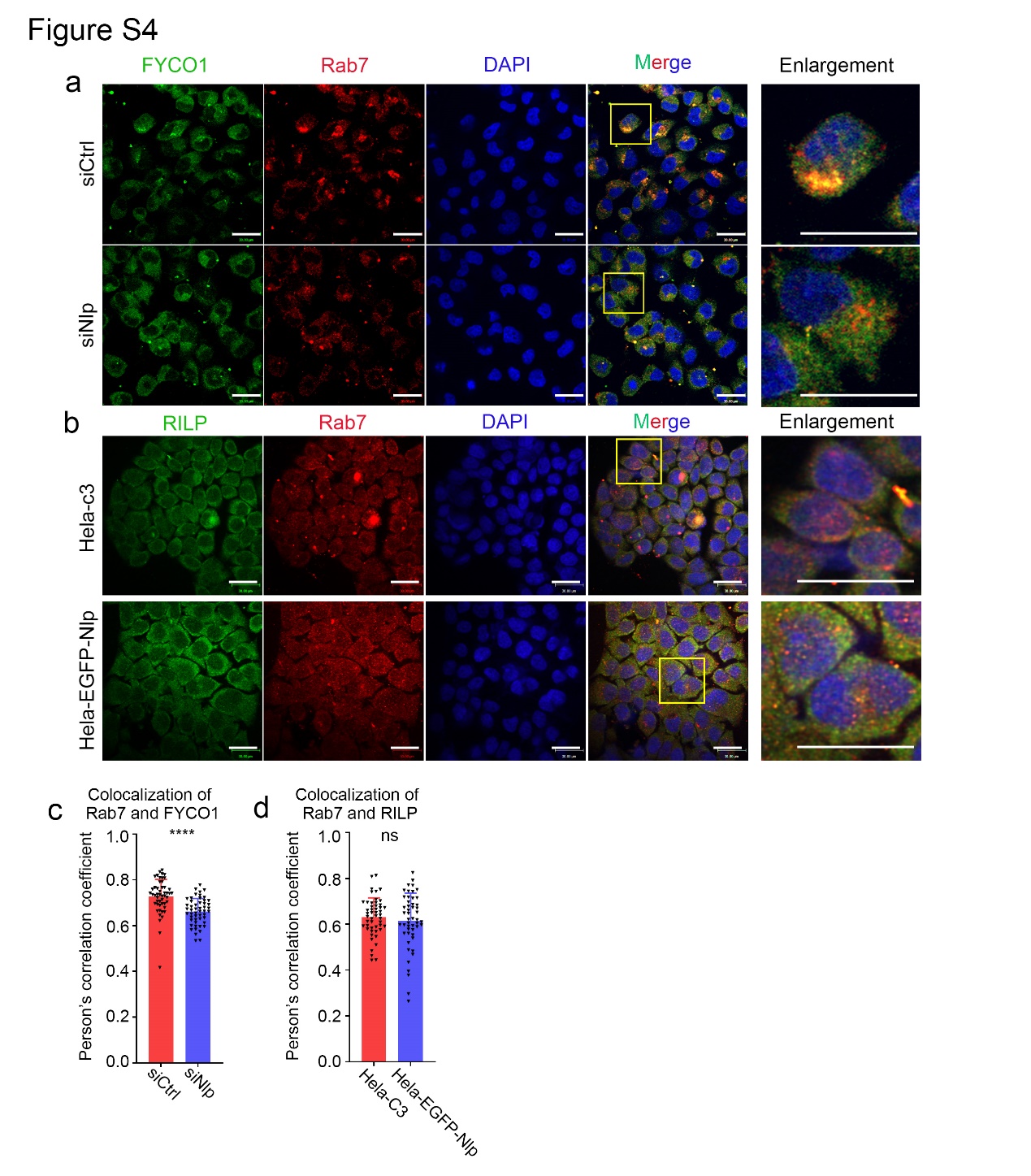


**Figure S4. Nlp influenced the colocalization of Rab7 and FYCO1 instead of Rab7 and RILP.** (a) Compared to control group (upper row), the colocalization of FYCO1 (green) and Rab7 (red) was decreased in Nlp knockdown NCI-H1299 cells (lower row). (c) Statistics data of colocalization of Rab7 and FYCO1, siCtrl and siNlp is compared. (b) In EGFP-Nlp overexpressed Hela cells and control, colocalization of Rab7 and RILP showed no significant difference. (d) Statistic data of colocalization of Rab7 and RILP, EGFP-Nlp overexpression and control is compared. Values are expressed as mean (bar) ± SD (error bar) for each group. (Scale bar for IF image is 30 μm. *p<0.05, **p<0.01, ***p<0.001, ****p<0.0001.)


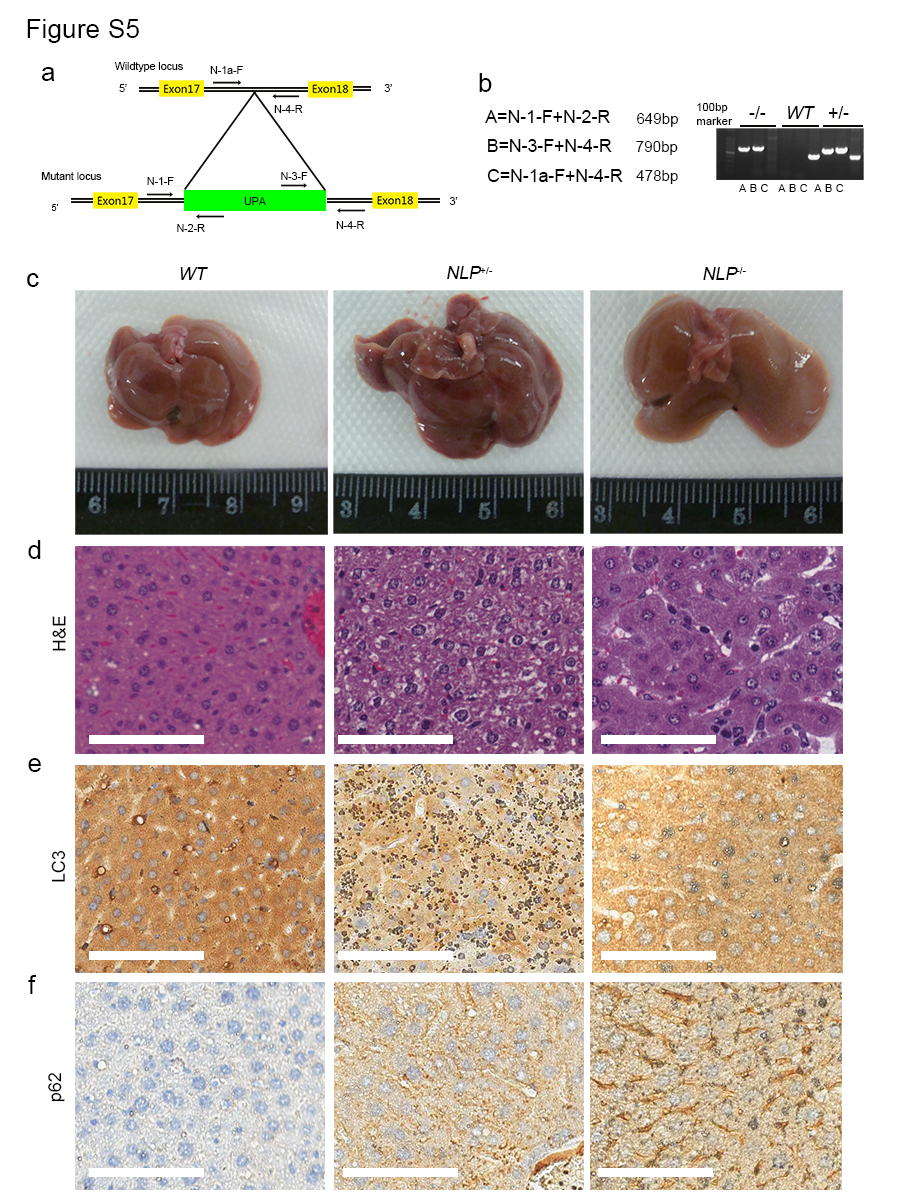


**Figure S5. Nlp deficient mice developed hepatomegaly induced by DMBA.** (a) Schematic representation of the structure of mice Nlp showing the position of UPA-Trap vector insertion. And specific primer sets were designed for genotyping PCR. (b) For vector insertion, the resulting PCR products would be 649 bp and 790 bp (for A and B respectively); for wild type, the resulting PCR products would be 478 bp (for C). The representative PCR band sets, and genotypes were shown. (c) Representative photos of tumor free liver dissected from mice of each genotype. (d-f) Representative images of H&E, IHC of LC3 and p62 of tumor free liver of each genotype. (Scale bar for H&E and IHC image is 100 μm.)


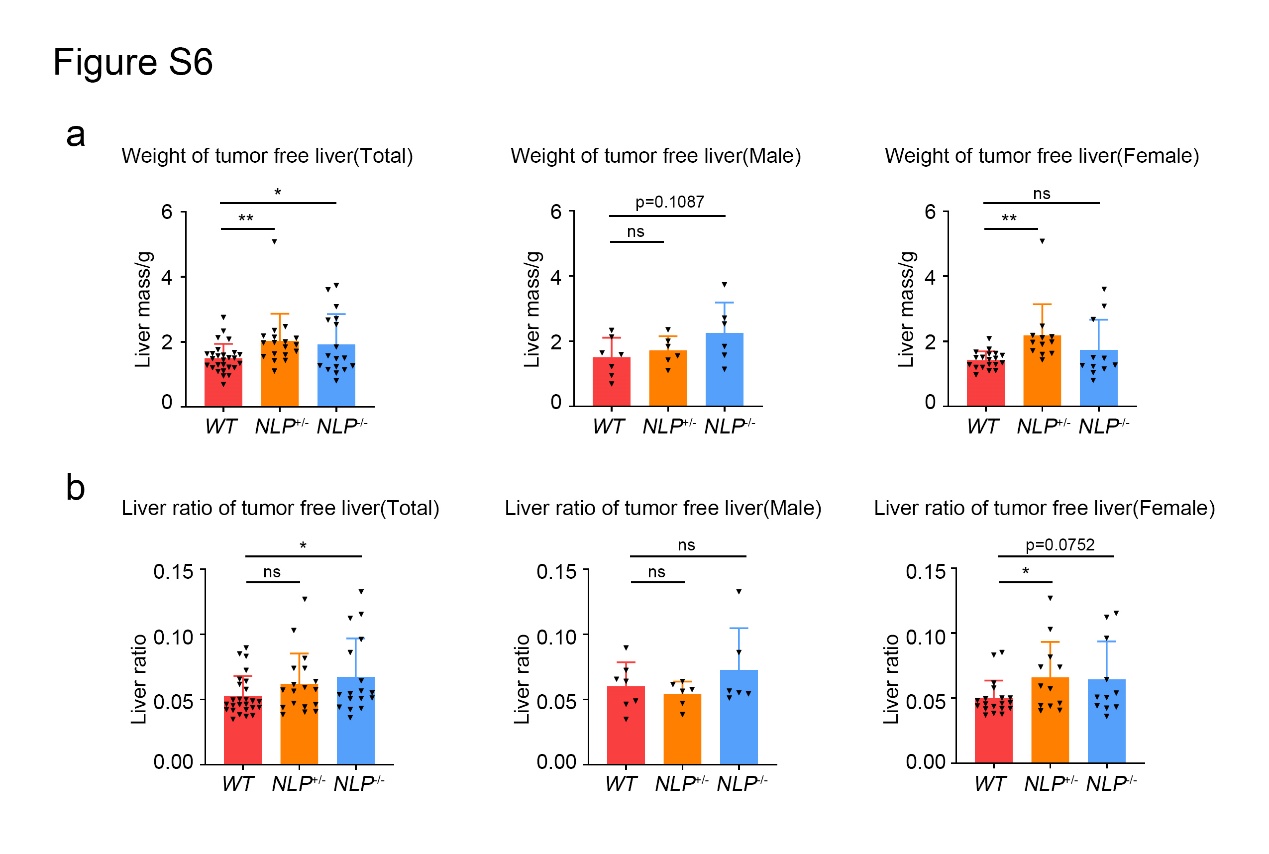


**Figure S6. The livers of Nlp deficient mice are larger than *WT* mice.** (a) Statistics of liver weight of tumor free liver from *WT*, *NLP*^+/-^ and *NLP*^-/-^ mice (total, male and female, respectively). (b) Statistics of liver-to-body weight ratio of tumor free liver from *WT*, *NLP*^+/-^ and *NLP*^-/-^ mice (total, male and female, respectively). All the organs were freshly dissected from mice and weighed following body weight was recorded. Student’s t-test was used to determine statistical significance. Values are expressed as mean (bar) ± SD (error bar) for each group. (*p<0.05, **p<0.01, ***p<0.001).
